# Acute obsessive symptoms: case report of a PANDAS-like syndrome in an adult patient.

**DOI:** 10.1192/j.eurpsy.2024.735

**Published:** 2024-08-27

**Authors:** Á. de Vicente Blanco, B. Orgaz Álvarez, P. Ibáñez Mendoza, M. Velasco Santos

**Affiliations:** ^1^Hospital Universitario La Paz, Madrid, Spain

## Abstract

**Introduction:**

Neuropsychiatric disorders can develop following a group A β-hemolytic streptococcal infection, through autoimmune inflammation of the nervous system. Sydenham’s chorea and PANDAS (Pediatric Autoimmune Neuropsychiatric Disorders Associated with Streptococcal Infection) are the two most well-known syndromes, primarily affecting children but rarely observed in adults.

**Objectives:**

Our aims are to contribute to the scientific understanding of adult PANDAS-like syndrome and provide a comprehensive literature review on the subject.

**Methods:**

Case report using clinical records and a non-systematic literature review.

**Results:**

A 24-year-old female presented to the emergency department with profound emotional distress triggered by intrusive thoughts of existential dread, accompanied by compulsive praying. She reported that these symptoms had commenced five days earlier. Two days prior to the onset of her obsessions, she had experienced a high fever, odynophagia, cough, and chills and received an empirical diagnosis of tonsillitis following a physical examination. She was prescribed antibiotics with good response. She revealed that she had experienced two prior episodes of similar anxiety and obsessions when she was approximately seven years old.

She developed acute obsessive thoughts, including doubts about the meaning of her life, and engaged in compulsive prayer and seeking reassurance from relatives. Notably, there were no signs of affective, dissociative, or psychotic disorders during her admission to the ED or in the preceding months. She reported suffering from anxiety, insomnia, and loss of appetite in the past five days but did not express any suicidal ideation.

Physical examination indicated mild laryngeal erythema, and laboratory tests showed non-specific signs of infection with no further significant findings. Symptoms were alleviated within a week, aided by treatment with benzodiazepines (lorazepam 1 mg/8h), and she did not require further psychiatric counselling.

**Conclusions:**

It is worth noting that adult patients can experience a PANDAS-like reaction after a streptococcal infection and may also undergo symptom relapse following new immunological challenges upon reinfection. The existence of a PANDAS spectrum has been postulated, encompassing various manifestations. Thus, when presented with acute obsessive symptoms, healthcare providers should consider this diagnosis, inquire about previous episodes, and conduct a comprehensive medical history and etiological assessment.

**Disclosure of Interest:**

None Declared

